# Association Between Thyroid Disorders and Colorectal Cancer Risk in Adult Patients in Taiwan

**DOI:** 10.1001/jamanetworkopen.2019.3755

**Published:** 2019-05-17

**Authors:** Abby L’Heureux, Daniel R. Wieland, Chien-Huan Weng, Yi-Huei Chen, Ching-Heng Lin, Tseng-Hsi Lin, Chien-Hsiang Weng

**Affiliations:** 1 Rural Medical Partners at Fallon Medical Center, Baker, Montana; 2 University of Arizona, Tucson; 3 Department of Immunology, Memorial Sloan-Kettering Cancer Center, New York, New York; 4 Department of Medical Research, Taichung Veterans General Hospital, Taichung, Taiwan; 5 Division of Hematology and Oncology, Department of Internal Medicine, Wuri Lin Shin Hospital, Taichung, Taiwan; 6 Department of Medicine, Chung Shan Medical University, Taichung, Taiwan; 7 Department of Family Medicine, Brown University Warren Alpert Medical School, Providence, Rhode Island; 8 Department of Family Medicine, Providence Community Health Centers, Providence, Rhode Island

## Abstract

**Importance:**

Thyroid hormones have been shown to affect several important pathways in cancer development, including colorectal cancer (CRC). Clinical studies examining the association between thyroid disorders and colorectal cancer have conflicting results and have predominantly involved white populations.

**Objective:**

To determine if a diagnosis of hyperthyroidism or hypothyroidism is associated with the risk of developing colorectal cancer in an East Asian population.

**Design, Setting, and Participants:**

This nationwide population-based case-control study was conducted from April 27, 2018, to November 8, 2018, using the Taiwanese National Health Insurance Research Database. Participants were adults (n = 139 426) either with a new diagnosis (between 2008 and 2013) of primary colorectal cancer without a history of cancer, or without cancer. Cases and controls were matched 1:1 by age, sex, and index date. Diagnosis of hyperthyroidism or hypothyroidism prior to the diagnosis of colorectal cancer (or the same index date in controls) was then determined.

**Main Outcomes and Measures:**

Risk differences in developing colorectal cancer among patients with a medical history of hyperthyroidism or hypothyroidism.

**Results:**

A total of 139 426 patients were included in the study, and 69 713 individuals made up each case and control group, which were both predominantly male (39 872 [57.2%]). The mean (SD) age for those with CRC was 65.8 (13.7) years and for those without CRC was 66.0 (13.6) years. Both hyperthyroidism (adjusted odds ratio [aOR], 0.77; 95% CI, 0.69-0.86; *P* < .001) and hypothyroidism (aOR, 0.78; 95% CI, 0.65-0.94; *P* = .008) were associated with a decreased risk of being diagnosed with colorectal cancer. An inverse association of rectal cancer was found among patients aged 50 years or older with a history of hypothyroidism despite treatment (aOR, 0.54; 95% CI, 0.39-0.74; *P* < .001). A history of hyperthyroidism in all age groups was associated with a lower risk of colon cancer (aOR, 0.74; 95% CI, 0.64-0.85; *P* < .001), with a stronger association seen among those younger than 50 years (aOR, 0.55; 95% CI, 0.36-0.85; *P* = .007).

**Conclusions and Relevance:**

In this study, hypothyroidism appeared to be associated with a lower risk of rectal cancer, whereas hyperthyroidism appeared to be associated with a lower risk of colon cancer. Because of this, biochemical in vivo research and epidemiologic studies appear to be needed to further clarify the nature of these associations.

## Key Points

**Question:**

Are thyroid disorders associated with colorectal cancer risk in an East Asian population?

**Findings:**

In this case-control study that included 139 426 adults in Taiwan with or without a diagnosis of primary colorectal cancer, both hyperthyroidism and hypothyroidism appeared to be associated with a statistically significantly decreased risk of colorectal cancer diagnosis.

**Meaning:**

Given these findings, it appears that biochemical in vivo research and epidemiologic studies are needed to further clarify the nature of the association found between thyroid disease and colorectal cancer and may potentially advance the therapies for colorectal cancer.

## Teaser

This population-based case-control study investigates whether a diagnosis of hypothyroidism or hyperthyroidism is associated with the development of colorectal cancer among adult patients in Taiwan.

## Introduction

Colorectal cancer (CRC) is an increasingly common disease with far-reaching consequences.^1^ It is the third most common cancer worldwide^2^ and the third leading cause of cancer-associated deaths in the United States, in which 1 in 22 men and 1 in 24 women are expected to develop the disease over the course of their lifetimes.^3^ Great efforts have been made to further characterize and understand the pathogenesis of CRC to develop new, more effective treatments for the disease.^4,5,6^ As is the case with many neoplastic processes, with CRC genetic and molecular alterations appear to be involved, leading to uncontrolled proliferation of these abnormal cells.^4^

Hormones have been identified as key factors associated with the development and evolution of many cancers and are being pursued as potential targets for therapy.^7,8,9,10,11^ This association may be most obvious within the realm of sex hormones and gynecologic cancers, but the role of trophic hormones, including the family of thyroid hormones, has come under greater scrutiny. Thyroid hormones, their nuclear and cellular surface receptors, and even antithyroid antibodies have been shown to be associated with several important pathways in cancer development.^5,6^

Studies in largely iodine-replete, white populations report the prevalence of hypothyroidism to be between 1% and 2%.^12^ The prevalence of hyperthyroidism ranges from 0.1% to 0.5% and is higher among females than males.^13^ Although numerous epidemiologic studies have detailed the association between thyroid dysfunction and cancer, to date relatively few studies have looked specifically at the association with CRC.^5,6,14,15,16,17,18,19^

Given that CRC is the fourth leading cause of death in Taiwan^20^ and that previous studies have involved predominantly non–East Asian populations, we conducted, to our knowledge, the first population-based study in Taiwan to elucidate the association between hyperthyroidism, hypothyroidism, and CRC. For this purpose, we used the Taiwanese National Health Insurance Research Database (NHIRD), what we believe is one of the most comprehensive health care databases in the world. The goal of this study was to uncover the association between thyroid dysfunction and CRC risk in an Asian population.

## Methods

This study was approved by the institutional review board of Taichung Veterans General Hospital, Taichung, Taiwan, which granted a waiver of informed consent because data are deidentified. Conducted from April 27, 2018, to November 8, 2018, this study followed the Strengthening the Reporting of Observational Studies in Epidemiology (STROBE) reporting guideline.

We designed a large, nationwide, population-based case-control study in which cases were defined as patients with a new diagnosis of CRC (diagnosed between January 1, 2008, and December 31, 2013) and no history of cancer, as recorded in the NHIRD. Controls, those without a CRC diagnosis, were randomly selected and matched 1:1 to the cases by age (by a margin of 1 month) and sex. We subsequently established the presence or absence of thyroid dysfunction prior to the diagnosis of CRC in the case group and, using the same index date as the matched case patient, in the control group.

### Taiwanese NHIRD

In 1995, the Taiwanese government established the National Health Insurance Program to provide coverage for most of the country’s population.^21^ The National Health Research Institute then created the NHIRD, a claims database overseen by the Taiwanese Department of Health. The NHIRD has multiple subset databases, including the Registry for Catastrophic Illness Patient Database and the Longitudinal Health Insurance Database. Because CRC is administratively assigned to be a catastrophic illness, a patient with a CRC diagnosis would apply and be registered within the Registry for Catastrophic Illness Patient Database. The Longitudinal Health Insurance Database includes 1 million randomly selected persons who represent the total Taiwanese insured population, which numbered approximately 23 460 000 by the end of 2013.^21^

### Case Selection and Case-Control Match

Case patients were identified from the NHIRD as having a new CRC diagnosis between 2008 and 2013. Diagnoses for CRC were registered using the *International Classification of Diseases, Ninth Revision, Clinical Modification* (*ICD-9-CM*) codes 153 and 154, which were cross-linked to the NHIRD. We used the presence of these codes, combined with a certificate of catastrophic illness, to identify the initial pool of 74 057 potential patient cases.

We excluded from the case group individuals whose age and sex were not known, those who were younger than 18 years or older than 120 years, and those who were deceased. We also excluded those with a diagnosis of malignant diseases before the CRC diagnosis, considering the possibility of metastasis to the colon or rectum. On the basis of these criteria, we identified a total of 69 713 cases.

A total of 69 713 policyholders were selected as controls in a 1:1 match with cases; they were randomly paired for age, sex, and the same index date (the month and year of CRC diagnosis in the case group) from the 2013 version (consistent with the study time frame) of the Longitudinal Health Insurance Database. Similar to our process for the case group, we excluded individuals from the control group whose age and sex were not known, who were younger than 18 years or older than 120 years, who had a diagnosis of other malignant diseases, and who were deceased before the index date.

### Hyperthyroidism, Hypothyroidism, and Other Adjustments

To identify patients with a thyroid disorder diagnosis, we searched for *ICD-9-CM* code 242 for hyperthyroidism and *ICD-9-CM* codes 243 and 244 for hypothyroidism. Additional criteria for inclusion were as follows: same diagnosis in at least 3 outpatient visits or 1 inpatient diagnosis followed by either another inpatient or outpatient visit with the same diagnosis. Patients with a diagnosis strictly from a single inpatient visit were excluded. The first diagnosis of a thyroid disorder must occur before the first CRC diagnosis in the case group or before the index date in the control group. We distinguished patients with acquired hypothyroidism from those with primary hypothyroidism by identifying the policyholders who first had a hyperthyroidism diagnosis (*ICD-9-CM* code 242) followed by hypothyroidism diagnoses (*ICD-9-CM* codes 244.0, 244.1, 244.2, and 244.3) (Figure).

**Figure.**
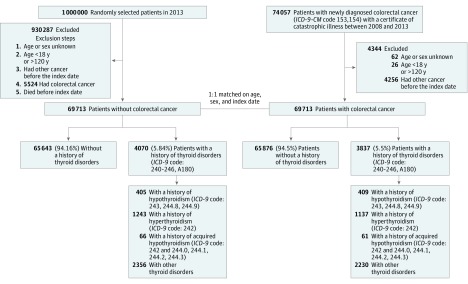
Flow Diagram of Participant Selection and Study Design No specific number is available for exclusions after random selection. Following the 5524 excluded who had colorectal cancer, a 1:1 match was performed for age, sex, and index date. ICD-9-CM indicates *International Classification of Diseases, Ninth Revision, Clinical Modification*.

We adjusted for a history of radioactive iodine treatment, diabetes, benign colorectal polyps, obesity, inflammatory bowel disease (IBD), and aspirin use. We also adjusted for age; sex; medication or surgical treatment for thyroid disease; colonoscopy within the 10 years prior to CRC diagnosis (or index date); and menopausal hormone therapy, including estrogen-only and estrogen-plus-progesterone treatments and labeling them as either ever used or never used.

### Statistical Analysis

We used the χ^2^ test to analyze categorical variables, to observe differences in clinical characteristics between the case and control groups. A conditional logistic regression analysis was applied to examine the association between thyroid disorders (hyperthyroidism, hypothyroidism, and acquired hypothyroidism) and the risk of developing CRC (CRC, colon cancer, and rectal cancer), in which we controlled for possible confounders (listed earlier). The confounders were known or were possible factors associated with CRC development or diagnosis. In the subgroup analysis, a logistic regression model was applied to examine the association between hyperthyroidism and hypothyroidism treatments and the risk of developing CRC, adjusted for sex; age; aspirin use; and history of IBD, obesity, benign polyps, diabetes, radioactive iodine treatment, hormone therapy, and colonoscopy. Statistical tests were all 2-sided, with *P* = .05 indicating the statistical significance level, and reported using 95% CIs and/or *P* values. All analyses were run using SAS, version 9.4 (SAS Institute Inc). Data were analyzed from May 3, 2018, to May 17, 2018.

## Results

A total of 139 426 patients were included in the study, and 69 713 individuals made up each of the case and control groups, which were both predominantly male (39 872 [57.2%]). The mean (SD) age for those with CRC was 65.8 (13.7) years and for those without CRC was 66.0 (13.6) years. Significant differences were found between the case group and control group in the proportion of those who ever used or never used various menopausal hormone therapy (19.7% vs 19.0%; *P* = .001), had a history of diabetes (14 506 [20.8%] vs 11 959 [17.2%]; *P* < .001), had a history of benign colorectal polyps (1200 [1.7%] vs 500 [0.7%]; *P* < .001), had a history of aspirin use (13 430 [19.3%] vs 12 416 [17.8%]; *P* < .001), and had a colonoscopy in the 10 years prior to CRC diagnosis or the same index date in the control group (53 970 [77.4%] vs 8282 [11.9%]; *P* < .001). In the case group, only 37 patients had received radioactive iodine treatment before their CRC diagnosis, whereas 33 in the control group had the same treatment before the index date. A total of 405 patients (0.6%) with hypothyroidism were identified in the control group, and 409 patients (0.6%) were identified in the case group. With regard to a hyperthyroidism diagnosis, 1243 patients (1.8%) were identified in the control group, and 1137 (1.6%) were identified in the case group (Table 1).

**Table 1.** Clinical Characteristics of Study Participants

Variable	No. (%)			*P* Value^a^
	Total (N = 139 426)	Without CRC (n = 69 713)	With CRC (n = 69 713)	
Age, mean (SD), y	65.9 (13.7)	65.8 (13.7)	66.0 (13.6)	**NA**
Sex				NA
Female	59 682 (42.8)	29 841 (42.8)	29 841 (42.8)	
Male	79 744 (57.2)	39 872 (57.2)	39 872 (57.2)	
Thyroid disorders				
No.	131 519 (94.3)	65 643 (94.2)	65 876 (94.5)	.07
With hypothyroidism	814 (0.6)	405 (0.6)	409 (0.6)	
With hyperthyroidism	2380 (1.7)	1243 (1.8)	1137 (1.6)	
With acquired hypothyroidism	127 (0.1)	66 (0.1)	61 (0.1)	
Others	4586 (3.3)	2356 (3.4)	2230 (3.2)	
Age <50 y				
No.	16 414 (95.2)	8216 (95.3)	8198 (95.1)	.04
With hypothyroidism	50 (0.3)	25 (0.3)	25 (0.3)	
With hyperthyroidism	332 (1.9)	176 (2.0)	156 (1.8)	
With acquired hypothyroidism	16 (0.1)	12 (0.1)	4 (0.0)	
Others	434 (2.5)	194 (2.2)	240 (2.8)	
Age ≥50 y				
No.	115 105 (94.2)	57 427 (94.0)	57 678 (94.4)	.02
With hypothyroidism	764 (0.6)	380 (0.6)	384 (0.6)	
With hyperthyroidism	2048 (1.7)	1067 (1.7)	981 (1.6)	
With acquired hypothyroidism	111 (0.1)	54 (0.1)	57 (0.1)	
Others	4152 (3.4)	2162 (3.5)	1990 (3.3)	
Hashimoto disease^b^				
No	139 273 (99.9)	69 624 (99.9)	69 649 (99.9)	.04
Yes	153 (0.1)	89 (0.1)	64 (0.1)	
Graves disease^c^				
No	138 516 (99.3)	69 243 (99.3)	69 273 (99.4)	.32
Yes	910 (0.7)	470 (0.7)	440 (0.6)	
History of aspirin use				
No	113 580 (81.5)	57 297 (82.2)	56 283 (80.7)	<.001
Yes	25 846 (18.5)	12 416 (17.8)	13 430 (19.3)	
History of IBD				
No	139 399 (100.0)	69 702 (100.0)	69 697 (100.0)	.34
Yes	27 (0.0)	11 (0.0)	16 (0.0)	
History of obesity^d^				
No	139 166 (99.8)	69 597 (99.8)	69 569 (99.8)	.08
Yes	260 (0.2)	116 (0.2)	144 (0.2)	
History of benign colorectal polyps^e^				
No	137 726 (98.8)	69 213 (99.3)	68 513 (98.3)	<.001
Yes	1700 (1.2)	500 (0.7)	1200 (1.7)	
History of diabetes^f^				
No	112 961 (81.0)	57 754 (82.8)	55 207 (79.2)	<.001
Yes	26 465 (19.0)	11 959 (17.2)	14 506 (20.8)	
History of radioactive iodine treatment^g^				
No	139 356 (99.9)	69 680 (100.0)	69 676 (99.9)	.63
Yes	70 (0.1)	33 (0.0)	37 (0.1)	
Medication treatment for thyroid disorder^h^				
No	136 790 (98.1)	68 367 (98.1)	68 423 (98.1)	.27
Yes	2636 (1.9)	1346 (1.9)	1290 (1.9)	
Thyroidectomy^i^				
No	138 538 (99.4)	69 260 (99.4)	69 278 (99.4)	.55
Yes	888 (0.6)	453 (0.6)	435 (0.6)	
Menopausal hormone therapy^j^				
No	112 456 (80.7)	55 976 (80.3)	56 480 (81.0)	.001
Estrogen only	12 974 (9.3)	6563 (9.4)	6411 (9.2)	
Estrogen plus progesterone	4942 (3.5)	2487 (3.6)	2455 (3.5)	
Both	9054 (6.5)	4687 (6.7)	4367 (6.3)	
Colonoscopy within 10 y of CRC diagnosis (or index date)^k^				
No	77 174 (55.4)	61 431 (88.1)	15 743 (22.6)	<.001
Yes	62 252 (44.6)	8282 (11.9)	53 970 (77.4)	

Abbreviations: CRC, colorectal cancer; IBD, inflammatory bowel disease; NA, not applicable.

^a^ *P* values calculated with *t* test, and χ^2^ test or Fisher exact test used for all other *P* values.

^b^ Hashimoto disease: *International Classification of Diseases, Ninth Revision, Clinical Modification* (*ICD-9-CM*) code 245.2; 3 outpatient visits or 1 inpatient hospitalization (excluding those with only 1 inpatient diagnosis); first diagnosis date before index date.

^c^ Graves disease: *ICD-9-CM* code 242.0; 3 outpatient visits or 1 inpatient hospitalization (excluding those with only 1 inpatient diagnosis); first diagnosis date before index date.

^d^ Obesity: *ICD-9-CM* code 278.0; 3 outpatient visits or 1 inpatient hospitalization; diagnosed 1 year prior to index date.

^e^ Benign colorectal polyps: *ICD-9-CM* codes 211.3, 211.4, and 569.0; 3 outpatient visits or 1 inpatient hospitalization; diagnosed 1 year prior to index date.

^f^ Diabetes: *ICD-9-CM* codes 250 and A181; 3 outpatient visits or 1 inpatient hospitalization; diagnosed 1 year prior to index date.

^g^ Radioactive iodine treatment: received at least 1 year prior to index date; excluded those within 1 year.

^h^ Medication treatment for thyroid disorder: used between the diagnosis of thyroid disease and index date.

^i^ Thyroidectomy: received between the diagnosis of thyroid disease and index date.

^j^ Menopausal hormone therapy: received at least 1 year prior to index date; excluded those within 1 year.

^k^ Colonoscopy: had 1 within 10 years prior to index date.

After adjusting for age; sex; aspirin use; and history of IBD, obesity, benign colorectal polyps, diabetes, radioactive iodine treatment, menopausal hormone therapy, and colonoscopy in the previous 10 years, we identified both hyperthyroidism and hypothyroidism as associated with a decreased risk of being diagnosed with CRC.

In all age groups, hypothyroidism was associated with a 22% lower risk of CRC (odds ratio [OR], 0.78; 95% CI, 0.65-0.94; *P* = .008) and a 45% lower risk of rectal cancer (OR, 0.55; 95% CI, 0.40-0.76; *P* < .001). Overall, hyperthyroidism was associated with a 23% lower risk of CRC (OR, 0.77; 95% CI, 0.69-0.86; *P* < .001) and a 26% lower risk of colon cancer (OR, 0.74; 95% CI, 0.64-0.85; *P* < .001). In general, for those who acquired hypothyroidism after hyperthyroidism treatment, no statistically significant change was found in risk of developing CRC (OR, 0.80; 95% CI, 0.70-0.82; *P* = .36).

Stratifying by age group revealed that the aforementioned decrease in CRC risk for patients with hypothyroidism diagnosis was associated with those aged 50 years or older with a rectal cancer diagnosis (aOR, 0.54; 95% CI, 0.39-0.74; *P* < .001); no other subgroup of patients with hypothyroidism diagnosis reached statistical significance. Hyperthyroidism in patients aged 50 years or older was associated with a lower risk of CRC (aOR, 0.78; 95% CI, 0.70-0.88; *P* < .001), but the inverse association was most profound in those younger than 50 years, specifically those with colon cancer (aOR, 0.55; 95% CI, 0.36-0.85; *P* = .007). No statistical significance was achieved concerning rectal cancer among those with a history of hyperthyroidism. The only subgroup with acquired hypothyroidism that achieved statistical significance comprised patients younger than 50 years, a small proportion of whom had colon cancer (aOR, 0.12; 95% CI, 0.02-0.94; *P* = .04).

In a related analysis of the association between autoimmune thyroid diseases and CRC, statistical significance was achieved concerning Hashimoto disease (aOR, 0.63; 95% CI, 0.41-0.97; *P* = .04) but not Graves disease (aOR, 0.87; 95% CI, 0.72-1.04; *P* = .12). Like hypothyroidism, Hashimoto disease in patients aged 50 years or older, when stratified by age, was associated with a lower risk of CRC (aOR, 0.60; 95% CI, 0.38-0.95; *P* = .03), more specifically rectal cancer (aOR, 0.34; 95% CI, 0.16-0.70; *P* = .004); statistical significance was not reached in the younger subgroup (Table 2).

**Table 2.** Adjusted Odds Ratio of Colorectal, Colon, or Rectal Cancer Associated With Thyroid Disorders

Variable	Colorectal Cancer		Colon Cancer		Rectal Cancer	
	Adjusted OR^a^ (95% CI)	*P* Value	Adjusted OR^a^ (95% CI)	*P* Value	Adjusted OR^a^ (95% CI)	*P* Value
Overall						
Without thyroid disorders	1 [Reference]	NA	1 [Reference]	NA	1 [Reference]	NA
With hypothyroidism	0.78 (0.65-0.94)	.008	0.92 (0.74-1.16)	.49	0.55 (0.40-0.76)	<.001
With hyperthyroidism	0.77 (0.69-0.86)	<.001	0.74 (0.64-0.85)	<.001	0.83 (0.69-1.00)	.05
With acquired hypothyroidism	0.80 (0.50-1.28)	.36	0.75 (0.43-1.30)	.30	0.95 (0.41-2.21)	.90
With Hashimoto disease	0.63 (0.41-0.97)	.04	0.78 (0.46-1.31)	.35	0.42 (0.21-0.86)	.02
With Graves disease	0.87 (0.72-1.04)	.12	0.80 (0.64-1.01)	.06	1.00 (0.75-1.34)	.99
Others	0.76 (0.70-0.82)	<.001	0.81 (0.74-0.89)	<.001	0.66 (0.57-0.76)	<.001
Age <50 y						
Without thyroid disorders	1 [Reference]	NA	1 [Reference]	NA	1 [Reference]	NA
With hypothyroidism	1.50 (0.72-3.11)	.28	1.44 (0.61-3.41)	.41	1.52 (0.39-5.97)	.55
With hyperthyroidism	0.67 (0.48-0.94)	.02	0.55 (0.36-0.85)	.007	0.92 (0.54-1.55)	.75
With acquired hypothyroidism	0.32 (0.06-1.66)	.17	0.12 (0.02-0.94)	.04	1.58 (0.18-13.5)	.68
With Hashimoto disease	0.87 (0.24-3.15)	.83	0.38 (0.08-1.79)	.22	2.66 (0.51-13.94)	.25
With Graves disease	0.90 (0.56-1.44)	.65	0.74 (0.40-1.37)	.34	1.25 (0.59-2.65)	.56
Others	0.92 (0.69-1.23)	.57	0.89 (0.61-1.30)	.54	1.00 (0.63-1.60)	.99
Age ≥50 y						
Without thyroid disorders	1 [Reference]	NA	1 [Reference]	NA	1 [Reference]	NA
With hypothyroidism	0.76 (0.63-0.91)	.004	0.90 (0.72-1.14)	.40	0.54 (0.39-0.74)	<.001
With hyperthyroidism	0.78 (0.70-0.88)	<.001	0.76 (0.66-0.88)	<.001	0.82 (0.68-1.01)	.06
With acquired hypothyroidism	0.90 (0.55-1.48)	.69	0.89 (0.50-1.60)	.70	0.92 (0.37-2.30)	.86
With Hashimoto disease	0.60 (0.38-0.95)	.03	0.82 (0.47-1.43)	.49	0.34 (0.16-0.70)	.004
With Graves disease	0.87 (0.71-1.05)	.14	0.81 (0.63-1.03)	.09	0.98 (0.71-1.35)	.92
Others	0.75 (0.69-0.81)	<.001	0.81 (0.73-0.89)	<.001	0.64 (0.55-0.74)	<.001

Abbreviations: NA, not applicable; OR, odds ratio.

^a^ Odds ratio was adjusted for sex; age; aspirin use; and history of inflammatory bowel disease, obesity, benign polyps, diabetes, radioactive iodine treatment, menopausal hormone therapy, and colonoscopy by logistic regression analysis.

In a subgroup analysis adjusting for medication and/or surgical treatment for thyroid disorders, no statistically significant difference was found between previous treatment for hypothyroidism (aOR, 0.93; 95% CI, 0.66-1.30; *P* = .66) and hyperthyroidism (aOR, 1.01; 95% CI, 0.82-1.25; *P* = .90) and the risk of CRC. Further stratifying hyperthyroidism treatment also revealed no significant difference between receiving a thyroidectomy (aOR, 1.40; 95% CI, 0.91-2.16; *P* = .13) and taking thyroid disorder medication (aOR, 0.97; 95% CI, 0.79-1.21; *P* = .82) and CRC risk (Table 3).

**Table 3.** Subgroup Analysis for Treatment-Adjusted OR of Colorectal Cancer Associated With TDs

Variable	Adjusted OR^a^ (95% CI)	*P* Value
Participants with hypothyroidism		
Without TD medications	1 [Reference]	NA
With TD medication^b^	0.93 (0.66-1.30)	.66
Participants with hyperthyroidism		
Without TD medications and thyroidectomy	1 [Reference]	NA
With TD medications^c^ or thyroidectomy^d^	1.01 (0.82-1.25)	.90
With thyroidectomy^e^	1.40 (0.91-2.16)	.13
With TD medication	0.97 (0.79-1.21)	.82

Abbreviations: NA, not applicable; OR, odds ratio; TD, thyroid disorder.

^a^ Odds ratio was adjusted for sex; age; aspirin use; and history of inflammatory bowel disease, obesity, benign polyps, diabetes, radioactive iodine treatment, hormone therapy, and colonoscopy by logistic regression analysis.

^b^ Hypothyroidism medication: levothyroxine sodium.

^c^ Hyperthyroidism medications: methimazole, propylthiouracil (did not include radioactive iodine treatment because it was adjusted separately).

^d^ Partial or total thyroidectomy.

^e^ If the patient received both medication and thyroidectomy, the patient would be classified as a surgical patient in this subgroup.

## Discussion

In this case-control study of 139 426 patients that used insurance claims data of nearly the entire population of Taiwan, we found that, after adjustment for many known risk factors, both hyperthyroidism and hypothyroidism were associated with a decreased risk of being diagnosed with CRC. When stratifying by age, the inverse association between hypothyroidism and rectal cancer appeared most pronounced in the subgroup of patients older than 50 years, whereas the inverse association between hyperthyroidism and colon cancer was most profound in patients younger than 50 years.

This study adds to the body of literature linking thyroid disease and CRC, which has accumulated some interesting, and seemingly contradictory at times, clinical results.^5,6,19,22,23,24,25,26^ One study found that, in general, patients with Graves disease have a higher risk of cancer.^24^ Another study seems to clarify this finding, reporting the increased cancer risk in hyperthyroid function to be associated with lung and prostate cancer but not with colon cancer.^26^ Boursi et al^5^ and Rennert et al^6^ both found a negative association between long-term thyroid hormone replacement with levothyroxine sodium and CRC risk. However, Rennert et al^6^ did not control for the preceding hypothyroidism, and Boursi et al^5^ found an overall higher risk of CRC among those with thyroid hormone replacement treatment for less than a year, untreated hypothyroidism, or hyperthyroidism. Shu et al^19^ found that, in patients hospitalized for Graves disease, risk of colon cancer was statistically significantly decreased. We hypothesized that these conflicting findings were largely associated with the complex interactions of thyroid hormones and their associated receptors with both normal and neoplastic colorectal tissues.

The biochemical association between thyroid hormones and colorectal cell differentiation, proliferation, tumorigenesis, and apoptosis is an involved and multifaceted process. The thyroid primarily secretes 2 hormones: thyroxine (T_4_) and triiodothyronine (T_3_).^27^ Their adverse effects are mediated by nuclear receptors *TRα1* and *TRβ1* and the plasma membrane integrin *αVβ3*, which has 2 thyroid hormone–binding sites (S1 and S2).^28^ Only T_3_ at physiological concentrations can bind to S1, triggering the phosphorylation and thus activation of the phosphatidylinositol-3-kinase pathway, which promotes cell proliferation and inhibits apoptosis.^29^ Activated by both hormones but primarily T_4_, S2 triggers the oncogenic extracellular signal-regulated kinase 1/2, facilitating a similar adverse effect while stimulating angiogenesis and the expression of fibroblast growth factor 2, components that are essential for rapid tumorigenesis.^30,31^

Alterations in thyroid hormone receptor *TRβ1* have been associated with colorectal adenomas and cancer, with data suggesting that *TRβ1* may play a tumor suppressor–type role in malignancy progression.^4^ Conversely, overexpression of thyroid hormone receptor *TRα1* appears to be associated with accelerated tumor appearance and progression.^32^ Similarly, thyroid hormone receptor interactor 13 has been shown to promote CRC cell proliferation, migration, and invasion^33^; T_4_ has been shown to promote β-catenin activation and cell proliferation in CRC^34^; and thyroid hormone binding of cell surface receptor *αVβ3* has been shown to lead to increased tumor cell proliferation and angiogenesis.^5^ *TRβ1* can also mediate the activation of the phosphatidylinositol-3-kinase pathway through interplay with T_3_ to ultimately promote the expression of transcription factor hypoxia-inducible factor 1’s α subunit; its genomic targets facilitate hypoxia resistance and angiogenesis found in metastasizing, rapidly growing tumors.^27,35^

Conversely, T_3_-activated *TRα1* is known to directly modulate the transcription of the β-catenin gene *CTNNB1* (MIM 116806), a proto-oncogene pivotal to the canonical Wnt signaling pathway, which has homeostatic and oncogenic cellular functions. Aberrant Wnt signaling is causally associated with colon cancer development.^31,34,36^

Colorectal cancer stem cells represent a small but important chemotherapy-resistant subgroup in CRCs that can generate the bulk of the tumor with their ability to divide asymmetrically and symmetrically.^37^ Thyroid hormone has also been shown to promote CRC stem cell depletion in CRC.^38^ A study by Catalano et al^39^ provides a potential mechanism for the inverse association seen among patients with hyperthyroidism; in their xenografts and in vitro models, T_3_-treated CRC stem cells had substantially reduced self-renewal capabilities; down-modulated Wnt signaling; decreased nuclear β-catenin buildup; and increased sensitivity to treatment, especially when D3 was knocked down. Intracellular T_3_ may have antitumorigenic properties as it induces differentiation among CRC stem cells. Studies also show that right-sided CRC and left-sided CRC share different molecular and genetic natures and have different levels of response to chemotherapy-or molecular-targeted- and immunotherapies as well as epidemiologic perspectives, such as incidence in different age groups and prognosis.^2,40,41,42,43,44,45,46^

Lin et al^31^ argued that T_4_’s role in carcinogenesis must be larger than T_3_ because of its greater binding affinity to *αVβ3* and naturally higher free-circulating levels. In addition, Lee et al^34^ provided evidence that T_4_’s nongenomic actions through binding with *αVβ3* also promoted nuclear β-catenin accumulation and that T_4_ in a dose-dependent manner promoted cell viability in CRC cell lines. Therefore, a potential mechanism may exist in which low free T_4_ from primary hypothyroidism could be protective against CRC by decreased interactions with the integrin.

### Strengths and Limitations

Strengths of the current study include the large sample size; the source of data representing nearly the entire population of Taiwan; the case-control design; and the adjustment for multiple CRC risk factors, including age; sex; aspirin use; and history of IBD, obesity, benign colorectal polyps, diabetes, iodine treatment, menopausal hormone therapy, and colonoscopy in the previous 10 years. By using insurance claims data from the NHIRD, we captured the desired data as completely and accurately as possible. The case-control nature of the study using stored, computerized data from the period in question nearly eliminates the potential for recall bias, and the large pool of data from which we pulled the cases and controls lends strength to the associations we found.

Limitations of the study include the homogeneous nature of the study population; lack of data on the compliance of thyroid hormone replacement; CRC type and stage; and variants of thyroid disease, such as subclinical hypothyroidism, thyroid adenoma, and toxic nodular goiter. Thyroid hormone replacement therapy seems to have protective properties against CRC, depending on the duration of treatment.^5^ We were not able to completely gather this information from the data, and this information could affect the associations we found. In addition, the use of *ICD-9-CM* codes that exactly matched hyperthyroidism and hypothyroidism may not have captured many cases along the spectrum of thyroid disease (eg, subclinical hypothyroidism, thyroid adenomas, and toxic nodular goiter), which have been suggested to have implications for cancer risk.^22,26^ We were not able to collect data on the type of CRC of the case patients. Colorectal cancer is believed to develop mostly through the adenoma-carcinoma sequence, but also either de novo or from flat adenomas.^4^ The pathways and signaling in these varying CRC types may differ considerably and therefore display different associations with hyperthyroid and hypothyroid states.

## Conclusions

In this case-control study, both hyperthyroidism and hypothyroidism appeared to be associated with a decreased risk of being diagnosed with CRC. To be more specific, hyperthyroidism was associated with a lower risk of colon cancer, and hypothyroidism was associated with a lower risk of rectal cancer. We hope these findings contribute to the body of literature on the association between thyroid disease and CRC and lead to changes in clinical practice for these diseases and potential advances in therapies for CRC. Our findings suggest that further biochemical in vivo research and epidemiologic studies are needed to clarify the nature of the association between thyroid disease and CRC and to apply this finding to clinical practice.
